# Interaction of Haloarchaeal Gas Vesicle Proteins Determined by Split-GFP

**DOI:** 10.3389/fmicb.2018.01897

**Published:** 2018-08-17

**Authors:** Kerstin Winter, Johannes Born, Felicitas Pfeifer

**Affiliations:** Microbiology and Archaea, Department of Biology, Technische Universität Darmstadt, Darmstadt, Germany

**Keywords:** gas vesicle proteins, split-GFP, protein–protein interaction, archaea, haloarchaea

## Abstract

Several extremely halophilic archaea produce proteinaceous gas vesicles consisting of a gas-permeable protein wall constituted mainly by the gas vesicle proteins GvpA and GvpC. Eight additional accessory Gvp are involved in gas vesicle formation and might assist the assembly of this structure. Investigating interactions of halophilic proteins *in vivo* requires a method functioning at 2.5–5 M salt, and the split-GFP method was tested for this application. The two fragments NGFP and CGFP do not assemble a fluorescent GFP protein when produced *in trans*, but they assemble a fluorescent GFP when fused to interacting proteins. To adapt the method to high salt, we used the genes encoding two fragments of the salt-stable mGFP2 to construct four vector plasmids that allow an N- or C-terminal fusion to the two proteins of interest. To avoid a hindrance in the assembly of mGFP2, the fusion included a linker of 15 or 19 amino acids. The small gas vesicle accessory protein GvpM and its interaction partners GvpH, GvpJ, and GvpL were investigated by split-GFP. Eight different combinations were studied in each case, and fluorescent transformants indicative of an interaction were observed. We also determined that GvpF interacts with GvpM and uncovered the location of the interaction site of each of these proteins in GvpM. GvpL mainly interacted with the N-terminal 25-amino acid fragment of GvpM, whereas the other three proteins bound predominately to the C-terminal portion. Overall, the split-GFP method is suitable to investigate the interaction of two proteins in haloarchaeal cells. In future experiments, we will study the interactions of the remaining Gvps and determine whether some or all of these accessory Gvp proteins form (a) protein complex(es) during early stages of the assembly of the gas vesicle wall.

## Introduction

**FIGURE 1:**
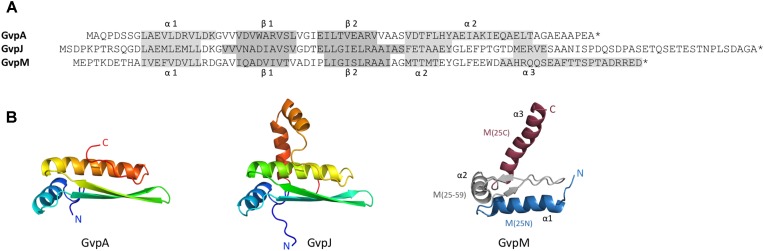
Sequence alignment of GvpA, GvpJ, and GvpM and predicted structural models of these proteins. **(A)** Sequence alignment of A, J, and M including the α-helices and β-strands proposed. These structures are highlighted in gray. **(B)** 3D-structural model of GvpA (Strunk et al., 2011) and homology models of GvpJ and GvpM calculated by I-Tasser server (Zhang, 2008; Roy et al., 2010; Yang et al., 2015) based on the *in silico* model of GvpA. In the case of GvpM, the helices α1 through α3, as well as the fragments M(25N), M(25–59), and M(25C) are indicated and color-coded. N and C designate the N- or C-terminus of these proteins.

Gas vesicles are proteinaceous structures synthesized by several bacteria and archaea, including the extremely halophilic archaeon *Halobacterium salinarum*. Gas vesicles enable the cells to float to the surface of the brine where light and oxygen concentrations are optimal for growth. The gas vesicle wall consists exclusively of aggregated proteins, and we are interested in investigating the protein–protein interactions required during their formation. *Hbt. salinarum* lives at salt concentrations of up to 5.3 M NaCl and uses the salt-in strategy to adapt to its salty environment. Isoosmotic potassium chloride concentrations are present in the cytoplasm, and the structure and function of most haloarchaeal proteins thus depends on salt. The gas vesicles are easy to isolate by lysis of the cells in water followed by centrifugation-enhanced flotation; they are stable in water or detergent solutions and only dissolve in 80% formic acid (Walsby, 1994; Belenky et al., 2004). Major constituent is the hydrophobic 8-kDa GvpA that forms antiparallel dimers aggregating into ribs running as low-pitch helix perpendicular to the long axis of the gas vesicle (Offner et al., 1998; Daviso et al., 2013). GvpA exhibits an α–β–β–α secondary structure with two α-helices separated by two β-strands (Sivertsen et al., 2010; Strunk et al., 2011). Due to its hydrophobic nature, a crystal structure of GvpA is not available. An *in silico* 3D-model of GvpA was obtained (**Figure 1B**) and challenged *in vivo* by analyzing the effect of single amino acid (aa) substitutions on gas vesicle formation in *Hfx. volcanii* ΔA+A_mut_ transformants (ΔA contains except for *gvpA* all *gvp* genes) (Strunk et al., 2011; Knitsch et al., 2017). Some mutations affect the formation of intact gas vesicles or influence the gas vesicle morphology. The single-layered protein wall is stabilized by the second structural protein GvpC attaching to the exterior surface. GvpC is not required for the formation of intact gas vesicles, since *Haloferax volcanii* ΔC transformants containing all *gvp* genes except for *gvpC* still produce gas-filled but odd-shaped structures (Offner et al., 1996). The haloarchaeon *Hfx. volcanii* is used for transformation studies since it offers a clean genetic background, is easy to transform, and grows much faster than *Hbt. salinarum* which contains at least two different *gvp* gene clusters (Pfeifer, 2012).

Gas vesicle formation involves 14 gas vesicle protein (*gvp*) genes arranged in two oppositely oriented transcription units, *gvpACNO* and *gvpDEFGHIJKLM* (Englert et al., 1990). Except for GvpC, GvpD, GvpE, GvpI, and GvpH all other Gvp proteins are essential (Offner et al., 2000). GvpD and GvpE are regulatory proteins affecting the *gvp* transcription (Plosser and Pfeifer, 2002; Zimmermann and Pfeifer, 2003). Immunological investigations as well as a MS/MS-based proteomic analyses of isolated gas vesicles demonstrate that most of the accessory Gvp proteins are present (Shukla and DasSarma, 2004; Chu et al., 2011). The accessory proteins GvpJ and GvpM exhibit sequence similarities of 48% (M-A), or 50% (J-A) to GvpA, and 60% similarity between J–M. Secondary structure predictions imply that GvpJ and GvpM also contain an α-helix near the N-terminus followed by a central region consisting of two β-strands including a β-turn (**Figure 1A**). In the case of GvpA, the β-strands most likely constitute the hydrophobic interior surface of the gas vesicle wall and prevent the precipitation of water molecules in the interior. The functions of GvpJ and GvpM, and of the other accessory Gvp are not yet known. They could be minor constituents of the gas vesicle wall, or act as scaffolding protein or chaperone to keep the hydrophobic GvpA in solution before incorporated in the wall. A crucial step in the formation of gas vesicles is the start of the Gvp aggregation. Since *gvpFGHIJKLM* is transcribed early in growth, the proteins encoded might be required in early stages of gas-vesicle assembly. All of these accessory Gvp are small, ranging in size from 9.2 (GvpM) to 32 kDa (GvpL). The involvement of GvpM in an early stage of gas vesicle formation was also proposed since point mutations in GvpM studied in ΔM+M_mut_ transformants resulted either in gas vesiculated (Vac^+^) or Vac negative cells (Tavlaridou et al., 2014).

Previously, we investigated putative protein–protein interactions of the accessory Gvp using His-tagged proteins bound to a Ni-NTA matrix to select their interacting partners. These analyses uncovered that GvpM is able to interact with GvpH, GvpJ, and GvpL, but not with GvpG (Tavlaridou et al., 2014). The studies involved the heterologous production of GvpX_His_ in *Escherichia coli* (i.e., under low salt concentrations), and isolation under denaturing conditions in 8 M urea using a Ni-NTA matrix (Tavlaridou et al., 2014). The GvpX_His_ proteins are refolded by dialysis against solutions containing decreasing urea and increasing salt concentrations up to 2.5 M, and refolded GvpX_His_ bound to Ni-NTA are then used to select other Gvp in cell extracts of *Hfx. volcanii* expressing the respective *gvp* gene under investigation. However, the Ni-NTA matrix also selects non-specifically additional proteins of *Hfx. volcanii* such as PitA and/or Cdc48d (Allers et al., 2010; Tavlaridou et al., 2014). Also, it is not clear whether the refolded proteins regain their native conformation.

To investigate protein–protein interactions in *Hfx. volcanii*, we tested the split-GFP method at high salt. This procedure has been used to investigate the interaction of proteins in bacteria and yeast (Ghosh et al., 2000; Magliery et al., 2005; Blakeley et al., 2012; Finnigan et al., 2016). The green fluorescent protein GFP is split between β-strands 7 and 8 into the N-terminal NGFP fragment containing the fluorophore and the C-terminal CGFP. Cells producing both fragments *in trans* do not assemble GFP and are thus not fluorescent. However, when both GFP fragments are fused to interacting proteins they assemble a fluorescent GFP. We used a modified version of the salt-stable smRS-GFP (Reuter and Maupin-Furlow, 2004) with an enhanced fluorescence and investigated protein–protein interactions in *Hfx. volcanii*. Compatible vector plasmids were used encoding Gvp fusions with the N-terminal or C-terminal fragment of this mGFP2. Our results confirmed the interactions M-L, M-H, and M-J, and we also determined that GvpF interacts with GvpM (M-F). In addition, fragments of GvpM were used to define the interaction sites of these four Gvp proteins with GvpM in further detail.

## Materials and Methods

### Strains and Cultivation Conditions

The *Escherichia coli* strains One Shot Top10 (Invitrogen by Life Technologies) and GM1674 (*dam^−^*) (Palmer and Marinus, 1994) were grown at 37°C overnight in Luria-Bertani broth. To select ampicillin-resistant clones, 100 μg/ml ampicillin was added. The haloarchaeon *Haloferax volcanii* WR340 was cultured in medium containing 3 M NaCl, 150 mM MgSO_4_, 50 mM KCl, 3 mM CaCl_2_ × H_2_O, 10 nM MnCl_2_, 25 mM Tris-HCl pH 7.2, 0.5% (w/v) tryptone, 0.3% (w/v) yeast extract, and 0.02% (w/v) histidine. For solid medium, 1.8% (w/v) agar was added. To select *Hfx. volcanii* transformants, 0.2 μg/ml novobiocin (for selection of pJAS35) and 6 μg/ml mevinolin (for selection of pWL_fdx_) were supplemented. Incubation was done at 42°C, and with the split-GFP expressing cells at 37°C or 37°C followed by 30°C as described in the “Results” section. Cultures on solid medium were incubated in humid atmosphere for 3–7 days, whereas liquid cultures were incubated 1–4 days with shaking at 180 rpm.

### Vector Construction and Transformation of *Hfx. volcanii*

The salt-stable smRS-GFP (Reuter and Maupin-Furlow, 2004) was improved in the fluorescence by the additional substitution F64L, and the resulting mGFP2 showed a 2.5-fold enhanced fluorescence (data not shown). Four vector plasmids were constructed to fuse the *ngfp*- or *cgfp*-portion of *mgfp2* at the 5′- or 3′-terminus of the respective *gvp* reading frame under investigation. The plasmids pJAS-NGFP-Nterm and pJAS-NGFP-Cterm are based on the shuttle vector pJAS35 (Pfeifer et al., 1994), and the plasmids pWL-CGFP-Nterm and pWL-CGFP-Cterm are based on pWL_fdx_ (Scheuch and Pfeifer, 2007) (**Supplementary Figure S1**). Both vectors occur in similar copy numbers per cell. Nterm describes the fusion of the respective *gfp* fragment at the 5′-terminus of *gvp* and Cterm the fusion at the 3′-end. GFP was split between amino acid residues 157 and 158, leading to NGFP (17.7 kDa) and CGFP (9.0 kDa). The synthetic oligonucleotides used to amplify the respective fragments (**Supplementary Table S1**) included also a linker sequence. The linker region in pJAS-derived vectors [(GGSGSGS)_2_] is 14 aa in size, and the linker region in the pWL vectors 16 aa [(GGSG)_4_]. Specific restriction sites were added leading to linker lengths of 15 to 19 aa (**Supplementary Figure S1**). The plasmid carrying *mgfp2* served as template for amplification of both fragments. The *BspH*I-*ngfp*-link-*Blp*I and the *Nco*I-link-*ngfp*-*Kpn*I fragments were inserted in the expression vector pJAS35, and the *Nco*I-link-*cgfp*-*Kpn*I and the *Nco*I-cgfp-link-*Kpn*I fragments in pWL_fdx_ (**Supplementary Figure S1**). In each case, the expression of the reading frames is driven by the strong *P_fdx_* promotor (Pfeifer et al., 1994) to yield similar and sufficient amounts of the fusion proteins. These vector constructions were all confirmed by DNA sequence analysis.

The *ngfp* or *cgfp* fragment was fused to the respective *gvp* reading frame encoding the Gvp under investigation. The *gvp* reading frames were amplified using the p-vac region of *Hbt. salinarum* (Offner et al., 1996) as template and synthetic oligonucleotides including the respective restriction sites (**Supplementary Table S1**). The *Nco*I-*gvp*-*Blp*I fragment was inserted in pJAS-NGFP-Nterm and pJAS-NGFP-Cterm (**Supplementary Figure S1**). For the insertion of *gvp* in pWL-CGFP-Cterm, the restriction sites *Nco*I and *BamH*I were used, and *BamH*I and *Kpn*I for the insertion in pWL-CGFP-Nterm. The correct fusion of each *gvp* to *ngfp* or *cgfp* was confirmed by DNA sequence determination. To overcome a restriction barrier in *Hfx. volcanii*, the plasmids were passed through the *E. coli* GM1674 (*dam^−^*) to obtain demethylated DNA. *Hfx. volcanii* was transformed simultaneously with the two vector plasmids as described previously (Pfeifer and Ghahraman, 1993), and the possession of both plasmids was confirmed by PCR and Western analysis.

### Western Analysis

The presence of N/CGFP-Gvp fusion proteins was confirmed by Western analysis. Total protein was isolated from 50 ml cultures in the exponential growth phase. The cells were harvested by centrifugation (2,370 × *g*, 30 min, 4°C) and re-suspended in 2–3 ml lysis buffer (2.5 M KCl, 50 mM MgCl_2_, 1 mM EDTA, 5% (v/v) glycerol, 50 mM Tris-HCl pH 8.0). Cell lysis was achieved by sonication on ice (2 × 2 min, Branson sonifier 250, 3 mm disruptor horn). The lysate was cleared by centrifugation (2,370 × *g*, 30 min, 4°C) and dialyzed against 10 mM Tris-HCl pH 7.2 for 2 h to eliminate salts. After dialysis, 20 μg of protein were separated by SDS-PAGE (Schagger and von Jagow, 1987) and transferred to a PVDF membrane (Roti^®^-Fluoro PVDF, Carl Roth) using the PerfectBlue^TM^ ‘Semi-Dry’-Blotter. The membrane was dried for 1 h, reactivated with 100% methanol, washed for 2 min in PBS (1.37 M NaCl, 27 mM KCl, 100 mM Na_2_HPO_4_, 20 mM KH_2_PO_4_ pH 7.4) and blocked for 1 h with Odyssey Blocking Buffer (LI-COR). The membrane was incubated overnight with the respective Gvp antiserum raised against GvpH, F, J, L, or M (Sartorius-Neef and Pfeifer, 2004; Tavlaridou et al., 2013). The membrane was washed four times for 5 min with PBS + 0.1% (v/v) Tween^®^ 20. Incubation with the secondary antibody IRDye 800CW (LI-COR) was done for 1–2 h and the membrane was washed four times for 5 min with PBS + 0.1% (v/v) Tween^®^ 20. To remove excessive Tween^®^ 20, the membrane was washed with PBS. The secondary antibody is coupled with a fluorophore detectable in the near-infrared range of 800 nm with an Odyssey Fc Imager (LI-COR).

### Quantification of Fluorescence

To demonstrate the protein–protein interaction *via* the assembly of NGFP and CGFP to a fluorescent protein, the fluorescence of the *Hfx. volcanii* transformants was quantified. In each case, 5-ml cultures were cultivated at 37°C to an optical density of 1–1.5, and the cultures were kept shaking at 30°C overnight. Two milliliters of these cultures were harvested by centrifugation (9,600 × *g*, 2 min, 20°C), washed with 1 ml basal salts (3 M NaCl, 150 mM MgSO_4_, 50 mM KCl), and re-suspended in 500 μl basal salts. Samples of 300 μl brought to OD_600nm_ 1 were analyzed in a 96-well plate and evaluated using the Fujifilm science lab image gauge ver. 4.24 software. Fluorescence measurements are given in light absorbing units (LAU) per mm^2^ (**Supplementary Table S2**). All experiments were performed with two biological samples and three technical replicates. The relative fluorescence (rf) was calculated using the formula given below and the standard deviation and the *p*-values were calculated using Student *t*-test

$$\mathrm{rf}=\frac{\mathrm{transformant}-\mathrm{untransformed WR340}}{\mathrm{untransformed WR340}}$$

### Fluorescence Microscopy

To investigate the cell fluorescence a Confocal Laser Scanning Microscopy (CLSM) was used. The transformants were grown to OD_600nm_ 1.5 and investigated. A Leica TCS SP5 II confocal microscope in combination with Leica application suite software was used for analysis. Image processing was done by the software Fiji.

## Results

In this study we investigated the protein–protein interactions of several accessory Gvp proteins involved in gas vesicle formation *in vivo* using a modified salt-adapted split-GFP.

### Adaptation of the Split-GFP Method to Haloarchaea

**FIGURE 2:**
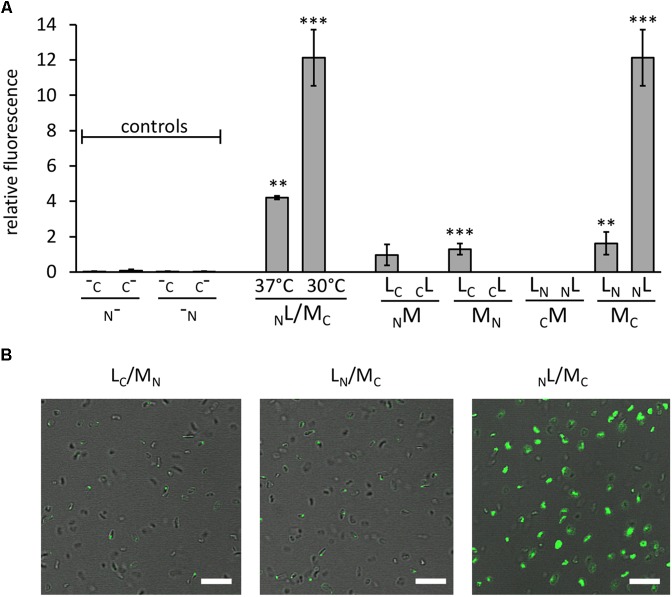
Interaction of GvpL and GvpM, as well as fluorescent *Hfx. volcanii* transformants. The fluorescence was determined in LAU/mm^2^ (**Supplementary Table S2**) and the relative fluorescence was calculated compared to the fluorescence of *Hfx. volcanii* WR340 cells. **(A)** Relative fluorescence of transformants carrying the “empty” vectors (controls; these encode GFP fragments, but lack the fusion to interacting proteins), and _N_L/M_C_ transformants grown at 37°C (OD 1 plus 1 day; final OD = 3.5) or at 37°C plus 1 day at 30°C (final OD = 2.5). In addition, the rf values of the eight L/M-N/CGFP transformants are shown. The Gvp proteins fused to NGFP or CGFP are indicated at the bottom of each graph. All experiments were performed with two biological samples and three technical replicates each. The significance was determined by Student *t*-test. ^∗∗∗^Significantly different from untransformed WR340, *P* < 0.001. ^∗∗^*P* < 0.01 **(B)** Fluorescence micrographs of transformants containing the L/M-N/CGFP fusions as indicated on top. The scale bare is 10 μm.

The modified green fluorescent protein mGFP2 (see “Materials and Methods”) was split between β-strands 7 and 8 to obtain the N-terminal fragment NGFP (residues 1–157) and the C-terminal CGFP (residues 158–239). The reading frames encoding these fragments were inserted in the compatible expression vectors pJAS35 (NGFP) and pWL_fdx_ (CGFP), initially providing a 7-aa linker between Gvp and N-/CGFP. Both plasmids occur in similar copy numbers per cell, and the expression of the inserted reading frames is driven by the ferredoxin promoter in both cases (Pfeifer et al., 1994; Scheuch and Pfeifer, 2007). The reading frames encoding the two interacting proteins GvpL (32 kDa) and GvpM (9.2 kDa) were used to test the method. However, fluorescent transformants were not observed (data not shown). To avoid a hindrance of the mGFP2 assembly, the 7-aa linker sequences were enlarged to 15 or 17 aa in the pJAS-derived vectors, and to 18 or 19 aa in the pWL vectors (**Supplementary Figure S1**). The resulting four plasmids allow the fusion of the reading frame of interest to *ngfp* or *cgfp* at the 3′- or the 5′-terminus. Four combinations of the “empty” vectors containing *ngfp* or *cgfp* but lacking a *gvp* reading frame were tested for an assembly of mGFP2 (**Figure 2A**, controls). None of these transformants indicated a higher fluorescence than *Hfx. volcanii* demonstrating that the self-assembly of mGFP2 did not occur (**Supplementary Table S2**).

**FIGURE 3:**
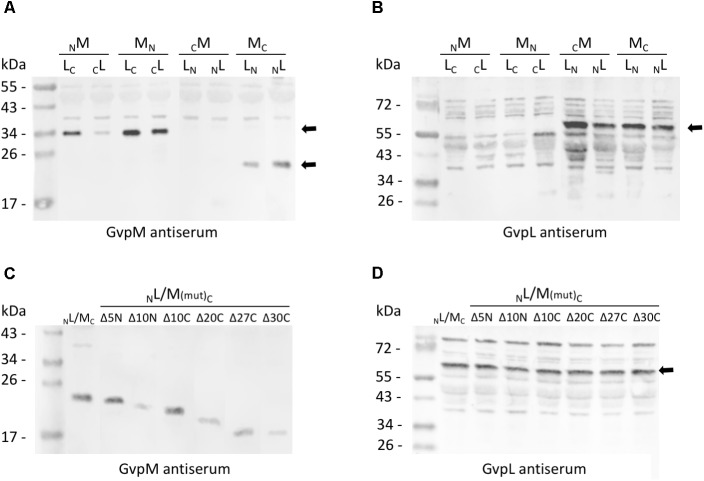
Western analysis of the various L/M transformants. Twenty micrograms of total protein were separated by SDS-PAGE, transferred to PVDF membranes and incubated with the antiserum raised against GvpM or GvpL. The second antibody was labeled with the fluorescence dye IRDye 800 CW (LI-COR) for detection. All blots are inverted to black and white. **(A)** Transformants carrying M/L-N/CGFP fusions and detection of GvpM using a GvpM antiserum. Arrows mark the M-NGFP and M-CGFP fusion proteins. **(B)** The same transformants as in **(A)** analyzed with the GvpL antiserum. The size of the L-NGFP fusions is marked by an arrow. **(C)** Detection of M_C_ and of M(mut)_C_ deletion variants in L/M transformants using the GvpM antiserum. **(D)** Detection of _N_L in the same transformants using the GvpL antiserum. The expected protein size is marked by an arrow.

Eight combinations of the plasmids carrying *gvpM* or *gvpL* fused to the *n/cgfp* fragments were tested in *Hfx. volcanii*. The resulting fusion proteins _NGFP_M, _CGFP_M, M_NGFP_, M_CGFP_, _NGFP_L, _CGFP_L, L_NGFP_, or L_CGFP_ carried NGFP or CGFP at the N- or C-terminus of GvpM or GvpL, and will be further described as _N_M, _C_M, M_N_, M_C_, _N_L, _C_L, L_N_, L_C_ for convenience. The fluorescence was initially measured in cells grown at 37°C, but the fluorescence emitted was relatively low (**Figure 2A** and **Supplementary Table S2**). To enhance the protein folding at lower temperatures, the cultures were grown to OD 1 at 37°C for 1 day to obtain sufficient cell mass, followed by incubation of the culture at 30°C overnight. This procedure increased the fluorescence signal threefold (**Figure 2A**) and demonstrated that the slower growth at 30°C helps folding and assembly of split-GFP. All eight L/M combinations were tested under the latter condition, and three of them yielded fluorescent transformants, i.e., _N_L/M_C_ (relative fluorescence, rf 12.1), L_N_/M_C_ (rf 1.6), and L_C_/M_N_ (rf 1.3) (**Figure 2A** and **Supplementary Table S2**). Inspecting the transformants by fluorescence microscopy determined that the entire cells of _N_L/M_C_ were fluorescent, whereas single fluorescent foci were observed with L_C_/M_N_ and L_N_/M_C_ transformants (**Figure 2B**), presumably causing the large difference in rf. The transformants were also investigated by Western analysis using an antiserum detecting GvpM or GvpL to ensure that the fusion proteins were produced (**Figure 3**). The _N_M, M_N_ and M_C_ proteins were well detectable, whereas _C_M was not found (**Figure 3A**). It is likely that the lack of fluorescence of L_N_/_C_M and _N_L/_C_M transformants was due to the undetectable amount of _C_M. In the case of GvpL, any of the NGFP-GvpL fusion proteins were observed, whereas the various CGFP-GvpL fusions were more difficult to detect since unspecific reactions of the GvpL antiserum occurred in the expected size range of 40–45 kDa (**Figure 3B**). Overall, an assembly of mGFP2 occurred mainly when N/CGFP was fused to the C-terminus of GvpM, whereas the N-terminal fusions of N- or CGFP yielded a low or undetectable fluorescence (**Figure 2A**). The latter results suggested that the N-terminal fusion might hinder the assembly of mGFP2, and that the N-terminal region of GvpM might be required for the GvpL interaction.

### Importance of the GvpM Termini for GvpL Interaction

**FIGURE 4:**
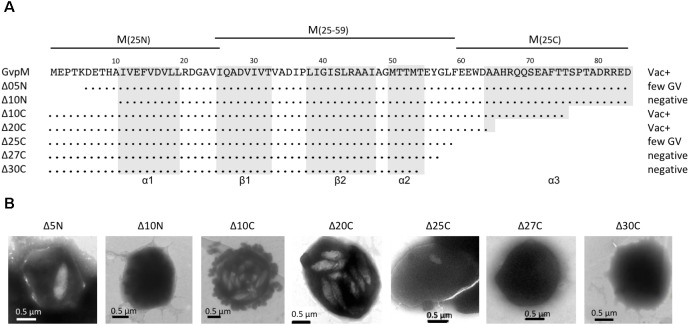
Deletion variants of GvpM and their Vac phenotype. **(A)** The 84-aa sequence of GvpM is given on top including the secondary structural elements α1, β1, β2, α2, and α3 shaded in gray. The different deletion variants are shown underneath. Dots refer to identical amino acids. The Vac phenotype observed with the respective *Hfx. volcanii* transformants is given on the right. Negative, gas vesicles were not observed; few GV, a single gas vesicle was found in a few cells, whereas other cells were Vac^−^; Vac^+^, fully gas-vesiculated cells. The fragments of GvpM used for the split-GFP analysis are indicated on top. **(B)** Transmission electron micrographs of various ΔM+MΔmut transformants. The size of the deletions at the N- or C-terminus of GvpM are indicated on top. Δ5N, Δ10N, and Δ10C were already described by Tavlaridou et al. (2014).

To determine the importance of the terminal regions of GvpM for gas vesicle formation and for the interaction with GvpL, different GvpM deletion variants were investigated (**Figure 4**). The two N-terminal deletion variants MΔ5N and MΔ10N, as well as the C-terminal deletion variant MΔ10C have been already tested for gas vesicle formation in ΔM+M_mut_ transformants (Tavlaridou et al., 2014). Construct ΔM contains except for *gvpM* all *gvp* genes, and GvpM produced *in trans* complements ΔM for gas-vesicle formation. ΔM+MΔ5N transformants produced a single gas vesicle per cell in a few cases, whereas ΔM+MΔ10N transformants are Vac negative, underlining the importance of the N-terminal region (Tavlaridou et al., 2014) (**Figure 4**). In contrast, a 10-aa deletion at the C-terminus results in gas-vesicle containing Vac^+^ ΔM+MΔ10C transformants. Further C-terminal deletions (up to 30 aa) were constructed and investigated for gas vesicle formation (**Figure 4**). Western analysis demonstrated that all GvpM deletion variants were stable and detectable (**Figure 3C**). Transmission electron microscopy showed that the ΔM+MΔ10C and ΔM+MΔ20C transformants contained many gas vesicles, whereas a few cells of the ΔM+MΔ25C transformants contained a single gas vesicle only, and ΔM+MΔ27C or ΔM+MΔ30C transformants were Vac negative (**Figure 4B**). These results implied that a large portion of the C-terminus of GvpM including helix α3 (aa 65–84) is not required for gas vesicle formation, whereas the N-terminal region is important.

**FIGURE 5:**
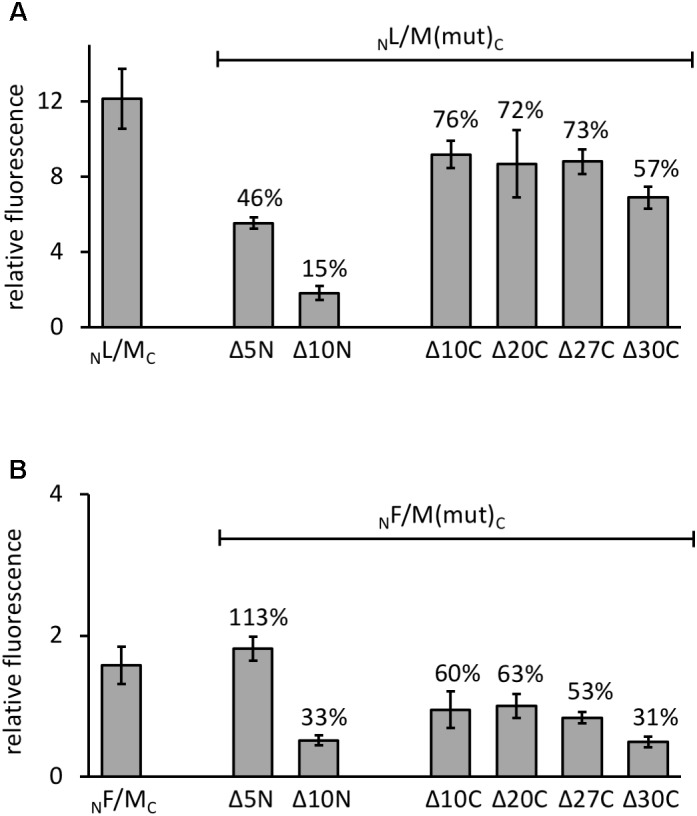
Interaction of GvpL and GvpF with GvpM deletion variants. The relative fluorescence was calculated in respect to the fluorescence obtained with *Hfx. volcanii* WR340. See **Supplementary Table S2** for rf values. Two biological samples and three technical replicates were analyzed in each case. The residual fluorescence compared to the positive control (_N_L/M_C_ or _N_F/M_C_) is given in percentage. **(A)** The _N_L/M_C_ and the respective deletion variants of GvpM are indicated at the bottom. **(B)** The _N_F/M_C_ and the respective deletion variants of GvpM used are indicated.

The various GvpM deletion variants were used to test the interaction with GvpL in the combination _N_L/M_C_ that showed the highest GFP fluorescence in *Hfx. volcanii* transformants. The transformants carrying the C-terminal deletions (_N_L/MΔ10C_C_ through _N_L/MΔ27C_C_) yielded 72–76% of the fluorescence obtained with _N_L/M_C_ transformants, and only the fluorescence of the _N_L/MΔ30C_C_ transformants was reduced to 57% (**Figure 5A**). These results implied that deletions at the C-terminus of GvpM had only a minor effect on the interaction with GvpL. The reduction to 57% with MΔ30C could be due to the relatively large deletion encompassing helix α3 and the loop between α2 and α3; this might affect the GvpM structure and also the L–M interaction. In the case of the N-terminal deletions, the fluorescence of _N_L/MΔ5N_C_ transformants was reduced to 46%, and in _N_L/MΔ10N_C_ transformants even to 15%, demonstrating a strong effect on the interaction with GvpL (**Figure 5A**).

### GvpL Interaction With Fragments of GvpM

**FIGURE 6:**
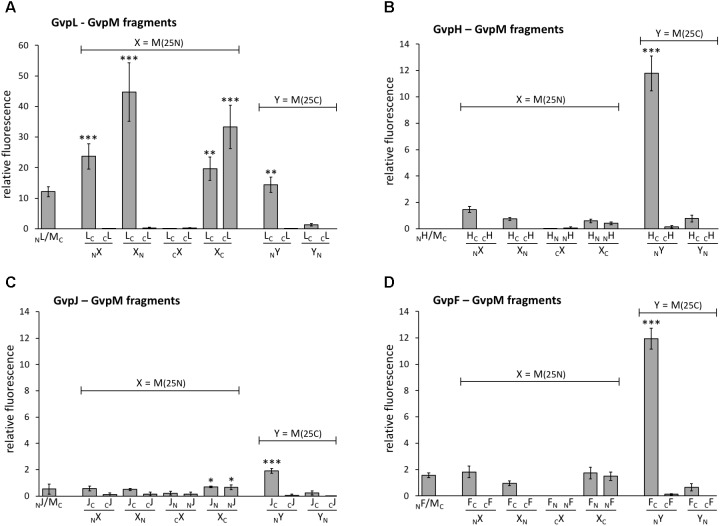
Interaction of GvpL, GvpF, GvpH, and GvpJ with fragments of GvpM. M(25N) contains the first 25 aa, and the C-terminal fragment M(25C) the last 25 aa of GvpM. The relative fluorescence was calculated in respect to the fluorescence obtained with the positive control. See **Supplementary Table S2** for LAU/mm^2^ and rf values. Two biological samples and three technical replicates were analyzed in each case. **(A)** L–M interaction; **(B)** H–M interaction; **(C)** J–M interaction; **(D)** F–M interaction. ^∗∗∗^Significantly larger compared to positive control, *P* < 0.001, ^∗∗^*P* < 0.01, ^∗^*P* < 0.05.

To challenge the hypothesis that the interaction of GvpL occurs in the N-terminal portion of GvpM, three fragments of GvpM were investigated, i.e., M(25N) encompassing the N-terminal 25 aa including helix α1, M(25–59) containing the central portion including the β-sheets plus α2, and the C-terminal fragment M(25C) with helix α3 (**Figure 4A**). Each of these fragments was fused to NGFP or CGFP at the N- or C-terminus and tested with the respective N/CGFP-GvpL fusions in *Hfx. volcanii* (**Figure 6A** and **Supplementary Table S2**). In the case of the N-terminal fragment M(25N), four of the eight combinations yielded highly fluorescent cells (rf 21–51), strongly supporting the idea that this fragment mediates the interaction with GvpL. In contrast, the eight combinations of the central fragment M(25–59) tested by split-GFP yielded no detectable fluorescence, and also the combinations including the C-terminal portion M(25C) showed no fluorescence except for L_C_/_N_M(25C) (rf 16) (**Supplementary Table S2** and **Figure 6A**). These results underlined that GvpL contacts GvpM preferentially in the N-terminal 25-aa. Compared to the interaction study using the full-length GvpM protein (rf 12), the fluorescence was much higher with M(25N) (rf 51), demonstrating that a smaller fragment is very useful to determine an interaction site.

### Interaction of GvpF, GvpH, and GvpJ With GvpM

To investigate additional interaction partners of GvpM, we studied the gas vesicle accessory proteins GvpF, GvpH and GvpJ by split-GFP. The interactions H-M and J–M were already demonstrated using His-tagged proteins bound to Ni-NTA matrices (Tavlaridou et al., 2014), but GvpF has not yet been investigated. In each case, the full-length GvpM was tested, but also the three fragments M(25N), M(25–59), and M(25C).

The 19.8-kDa GvpH is able to prevent the aggregation of GvpM in *Hfx. volcanii* transformants and might act as chaperone (Tavlaridou et al., 2014). Analyses using His-tagged GvpH demonstrated the H–M interaction, but no fluorescence was detectable when the entire GvpM and GvpH were tested in all eight combinations by split-GFP (**Figure 6B** and **Supplementary Table S2**). A low fluorescence (up to rf 1.5) occurred when fragment M(25N) was used, and no fluorescence was detectable in all combinations of the central portion M(25–59). However, the C-terminal M(25C) fragment yielded a high fluorescence in H_C_/_N_M transformants (rf 12; **Figure 6B**), suggesting an interaction of GvpH with the C-terminal portion of GvpM. In the case of the GvpM-related GvpJ (12 kDa), a very low fluorescence was observed with the _N_J/M_C_ transformants carrying the full-length GvpM (**Figure 6C**). A similarly low fluorescence was observed in all cases when the N-terminal fragment M(25N), or the central fragment M(25–59) were tested with GvpJ (**Figure 6C** and **Supplementary Table S2**). The C-terminal fragment M(25C) yielded a slightly enhanced fluorescence (rf 1.9) with J_C_/_N_M(25C) transformants. Analyses using His-tagged GvpJ demonstrated the J–M interaction, and the results presented here suggested that GvpJ might contact the C-terminal portion of GvpM (**Figure 6C**).

Investigating the 23.7-kDa GvpF for interaction with GvpM yielded a low fluorescence (rf 1.6) in _N_F/M_C_ transformants, and also with some combinations of M(25N) (rf 1.5–1.8, **Figure 6D** and **Supplementary Table S2**). No fluorescence was detectable with the central region M(25–59), but a high fluorescence (rf 12) was obtained with F_C_/_N_M(25C) transformants (**Figure 6D** and **Supplementary Table S2**). The latter result implied that GvpF interacts with the C-terminal portion of GvpM. To support these results, the N- and C-terminal deletion variants of GvpM were tested with the split-GFP method. Using MΔ5N for the investigation of the F–M interaction, the fluorescence was very similar to GvpM wild type (**Figure 5B**, 113%). The _N_F/MΔ10N_C_ transformants yielded a strongly reduced fluorescence (33% of the GvpM wild type) implying that the sequences deleted are involved in the interaction with GvpF. All transformants harboring a C-terminal deletion in GvpM (MΔ10C through MΔ27C) showed reductions to 63 and 53% of the wild type, and the fluorescence of the _N_F/M_Δ30C_ transformants was reduced to 31% (**Figure 5B**). Overall, these results supported the hypothesis that the interaction F–M mainly occurs in the C-terminal portion of GvpM.

In summary, our data implied that the accessory proteins GvpF, GvpH, and GvpJ interact predominantly with the C-terminal portion of GvpM. In each case, the highest fluorescence was achieved in the combination F_C_/-, H_C_/-, or J_C_/_N_M(25C), i.e., when NGFP was fused to the N-terminus of M(25C) and CGFP to the C-terminus of the accessory protein tested.

## Discussion

Investigations of the (dynamic) protein–protein interactions are important to understand the protein aggregations that occur during the formation of gas vesicles in haloarchaea. The split-GFP method has been applied in bacteria and yeast to analyze the interactions of proteins, e.g., involved in cell division (Blakeley et al., 2012; Finnigan et al., 2016). Since GFP is very stable once assembled from the fragments NGFP and CGFP, even low affinities of the interacting proteins are detectable (Magliery et al., 2005). Haloarchaea contain molar concentrations of potassium in the cytoplasm, and the split-GFP method was adapted using the modified salt-stable mGFP2 protein that carries the additional substitution F64L. Four vectors are now available to engineer the N- or C-terminal fusion of a protein of interest with one of the fragments of mGFP2. These vectors are based on the two compatible plasmids pJAS35 and pWL_fdx_ (Pfeifer et al., 1994; Scheuch and Pfeifer, 2007). In both cases, the inserted reading frames are expressed under the control of the ferredoxin promoter and yield sufficient and similar amounts of the fusion proteins. Linker regions of 15- to 19-aa were useful and supported the assembly of mGFP2. The folding and/or assembly was enhanced by lowering the growth temperature to 30°C. *Hfx. volcanii* grows rather slow at 30°C, since the optimal cultivation temperature is 42°C. Thus, the transformants were grown at 37°C to yield enough cell mass, and the cells were then incubated for 16 h at 30°C to assist mGFP2 assembly. This procedure yielded a threefold higher fluorescence of the cells compared to cells grown at 37°C only.

### GvpL Interacts With the N-Terminal Fragment of GvpM

**FIGURE 7:**
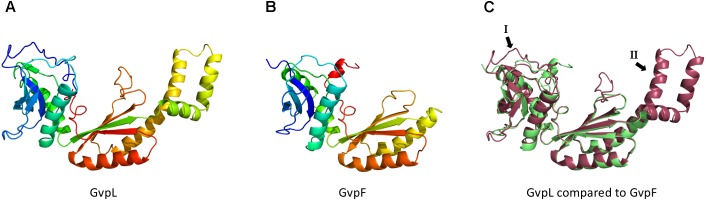
Models of the 3D-structures of GvpL and GvpF. Both 3D-models were obtained by homology modeling using the 3D-crystal structure of GvpF derived from *Microcystis aeruginosa* (Xu et al., 2014). The structural models were calculated by I-Tasser server (Zhang, 2008; Roy et al., 2010; Yang et al., 2015) **(A)** Homology model of GvpL. **(B)** Homology model of GvpF. **(C)** Alignment of the homology models of GvpL (red) and GvpF (green). The two major structural differences are labeled I or II and marked by arrows. The inserts I and II are also labeled in the alignment of the GvpF and GvpL sequences presented in **Supplementary Figure S2**.

GvpM and GvpL were used to demonstrate the function of the split-GFP method. GvpM is a hydrophobic, small protein of 9.2 kDa with sequence and structural similarities to the major gas vesicle protein GvpA (**Figure 1**), whereas GvpL is with 32 kDa relatively large (**Figure 7A**). Eight combinations of the four different N/CGFP fusion variants were tested in *Hfx. volcanii*. The highest fluorescence was obtained in the combination _N_L/M_C_ (i.e., N-terminal fusion of NGFP to GvpL and C-terminal fusion of CGFP to GvpM), and to a less extent with L_N_/M_C_, and L_C_/M_N_, whereas all other combinations did not result in a detectable GFP fluorescence. Thus, it is important to analyze the different combinations of N/CGFP fusions, since GFP assembly of the two fragments depends on physical constraints of the interacting proteins. The highly fluorescent _N_L/M_C_ transformants contained the assembled mGFP2 distributed in the cells, whereas other transformants harbored aggregated mGFP2 as a single fluorescent focus per cell, presumably caused by an aggregation of GvpM. The N- or C-terminal fragment of GvpM lacking the hydrophobic central portion always yielded fully fluorescent cells when tested by split-GFP (data not shown). Since mainly C-terminal fusions of the N/CGFP-fragments to GvpM yielded fluorescent cells, we hypothesized that the N-terminus of GvpM was involved in the L–M interaction. Testing the N-terminal 25-aa of GvpM confirmed that the contact site is located here. In addition, another less distinct interaction site might be located in the C-terminal portion of GvpM. The smaller fragments were excellent interaction partners for GvpL, since the structural constraints for the assembly of mGFP2 are lower. This was already shown by testing small leucine-zipper regions of a transcriptional regulator in *E. coli* for an interaction (Magliery et al., 2005).

The hypothesis that GvpL contacts GvpM mainly near the N-terminus was also supported by the analysis of GvpM deletion variants. Variants that incurred deletions of up to 27-aa at the C-terminus yielded a similar fluorescence compared to the full-length GvpM, whereas the N-terminal GvpM deletion variant MΔ10N yielded a residual fluorescence of only 15% underlining that the lack of these sequences affected the interaction with GvpL. The deletion encompasses the N-terminal sequence up to helix α1, and it will be interesting to test point mutations in order to define the interaction site of GvpL more precisely.

### GvpF, GvpH, and GvpJ Interact With the C-Terminal Fragment of GvpM

The C-terminal fragment of GvpM comprising the helix α3 appeared to interact with the accessory proteins GvpF and GvpH, and presumably with GvpJ. A high fluorescence was observed in the combinations F_C_/_N_M(25C) and H_C_/_N_M(25C) and to a less extent in J_C_/_N_M(25C), whereas the central portion of GvpM yielded no fluorescent transformants. It is interesting to note that GvpF (23.9 kDa) and GvpL (32 kDa) have very similar 3D-structures. A crystal structure of GvpF derived from the cyanobacterium *Microcystis aeruginosa* is available and shows two structurally distinct domains displaying an α+β structure (Xu et al., 2014). This crystal structure was used for the homology modeling of the haloarchaeal GvpF and GvpL (**Figure 7**). Both 3D-models are similar and contain two domains with α+β folds (**Figure 7C**). Nevertheless, the similarity of their amino acid sequences is not very high (35%) (**Supplementary Figure S2A**). Our analysis on the F–M and L–M interactions implied that both proteins bind to different portions of GvpM, since GvpL showed a high affinity to the N-terminal portion, and GvpF to the C-terminal portion of GvpM. It is possible that GvpL and GvpF bind simultaneously to GvpM, and a possible complex formation should be investigated. The interaction of GvpF with the C-terminal portion of GvpM is somewhat surprising since this region is not required for gas vesicle formation; most of this region could be deleted without the loss of gas vesicle formation. A sequence alignment of GvpM derived from the p-vac region (pGvpM was investigated here) and cGvpM encoded by the second gas vesicle region c-vac of *Hbt. salinarum* shows that the last 11 aa of pGvpM are not found in cGvpM (**Supplementary Figure S2B**). This observation suggests that the binding site(s) of the three accessory Gvp proteins might be located further upstream, possibly in the loop between the helices α2 and α3. Also here, point mutations should be tested to determine the interaction sites more precisely.

The 19.8-kDa GvpH prevents the aggregation of GvpM as demonstrated with M_GFP_+H transformants in comparison to M_GFP_ transformants using a fluorescent GvpM–GFP fusion (Tavlaridou et al., 2014). The M_GFP_ transformants contain fluorescent foci indicative of aggregated M_GFP,_ whereas the M_GFP_+H transformants are fully fluorescent. Thus, we hypothesized that GvpH keeps GvpM in solution. However, GvpH is not required for gas vesicle formation, since ΔH transformants form gas-filled structures, but these gas vesicles are fragile and collapse into ribs when treated with uranyl-acetate for transmission electron microscopy (Offner et al., 2000). Previous protein interaction studies showed that GvpH is selected by GvpM_His_ bound to Ni-NTA (Tavlaridou et al., 2014). Our analysis by split-GFP showed that mainly the C-terminal fragment M(25C) interacted with GvpH (and to a less the N-terminal portion of GvpM) implying that GvpH preferentially binds to the C-terminal portion of GvpM.

GvpJ (12 kDa) is a small, hydrophobic protein related to GvpA and GvpM and structural modeling suggests a similar 3D-structure (**Figure 1B**). The analysis of GvpJ by split-GFP detected a low fluorescence in transformants harboring the full-length GvpM or fragment M(25N). Only transformant J_C_/_N_M(25C) yielded a nearly fourfold higher fluorescence than transformants harboring the full-length GvpM. However, the relative fluorescence was much lower compared to the fluorescence obtained for the F–M and H–M interaction (**Figure 6C** and **Supplementary Table S2**). The low fluorescence observed with the J–M interaction is in contrast to the strong selection of GvpJ from a *Hfx. volcanii* lysate by GvpM_His_ bound to Ni-NTA (Tavlaridou et al., 2014). It is possible that the fragmentation of GvpM disturbed the contact sites (or the protein fold) of GvpJ, since the loop region between β2 and α2 was disrupted (**Figure 4A**). Also, it is possible that the interaction requires not a consecutive aa sequence, but amino acids that occur in close location at the surface. In addition, the low fluorescence could be due to the hydrophobic nature of GvpJ. The aggregation could influence the availability of GvpJ for the interaction with GvpM.

In summary, we demonstrated that the interaction of haloarchaeal proteins can be studied by split-GFP *in vivo*. Three of the protein pairs analyzed confirmed previous results using His-tagged proteins bound to Ni-NTA matrices *in vitro*. The advantage of the split-GFP method is that the analysis is conducted *in vivo* without having to isolate the salt-adapted proteins under low-salt concentrations. Our experiments uncovered that the accessory protein GvpF interacts with GvpM, and we were able to confine the interaction sites of all accessory Gvp tested to the N- or the C-terminal portion of GvpM. GvpL interacted predominantly with the N-terminal region of GvpM, whereas GvpF, GvpH, and GvpJ preferred the C-terminal portion of GvpM, raising the question whether the three proteins bind simultaneously or consecutively to GvpM during gas vesicle formation. The *gvpFGHIJKLM* genes are co-transcribed in *Hbt. salinarum* leading to a consecutive synthesis starting with GvpF and concluding with GvpL and GvpM. It is possible that all these accessory proteins form (or are part of) a larger protein complex. The split-GFP method will be applied to determine additional interactions between the accessory Gvp and also with GvpA. In addition, we will investigate whether the accessory Gvp proteins form a larger protein complex during gas-vesicle assembly, but this requires different methods.

## Author Contributions

KW and FP planned the study, discussed the results, and wrote the manuscript. KW performed the analysis. JB designed mGFP2 used for the construction of split-GFP. All authors approved the final manuscript.

## Conflict of Interest Statement

The authors declare that the research was conducted in the absence of any commercial or financial relationships that could be construed as a potential conflict of interest.
