# Supplementary material for: Interaction of Haloarchaeal Gas Vesicle Proteins Determined by Split-GFP
Source: Front Microbiol. 2018 Aug 17;9:1897. doi: 10.3389/fmicb.2018.01897 (PMC6107691; doi:10.3389/fmicb.2018.01897)
Supplement: Supplementary file 1 [file Presentation_1.PDF]

Supplementary Material

Interaction of Haloarchaeal Gas Vesicle Proteins Determined by Split-GFP

Kerstin Winter[1], Johannes Born[1], and Felicitas Pfeifer[1*]

[1]Microbiology and Archaea, Department of Biology, Technische Universität Darmstadt, Darmstadt, Germany

*** Correspondence:**
Prof. Dr. Felicitas Pfeifer
pfeifer@bio.tu-darmstadt.de

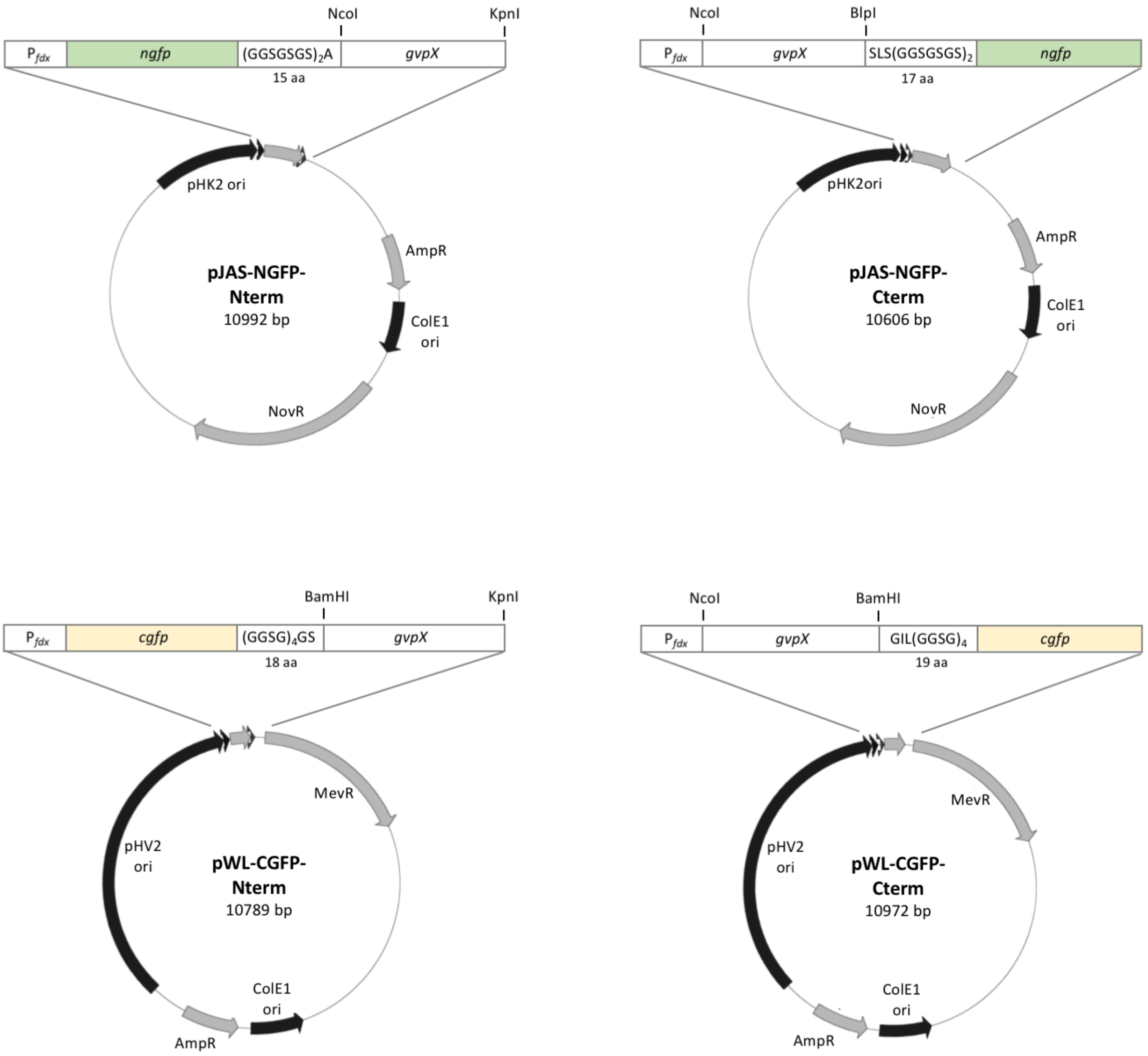

Figure S1. The four expression vectors used for the construction of N/CGFP fusions. The vectors are based on the compatible haloarchaeal expression vectors pJAS35 (Pfeifer et al., 1994) and pWL$_{fdx}$ (Scheuch et al., 2004), both expressing the inserted reading frames under the control of the ferredoxin promoter.

A

```
                                    I
GvpF (M. aeruginosa)    MTVGL--------------------YLYGIFPEPIPDGLVL--QGIDNEPVHSEMIDGFSFLYSAAH----KEKY
GvpF (Hbt. salinarum)   MTENL--------------------YTYGIIEQEDLELDVEGVAGAEQ--VYPVDYKTLSAVVSDIDTTDPERTD
GvpL (Hbt. salinarum)   MTDHRPSPEEEQTTANEERTVSNGRYLYCVVDTTSSESATLSTTGVDDNPVYVVEADGVGAVVHDCETVYETEDL

GvpF (M. aeruginosa)    LASRRYLICHEKVLETVMEAGFTTLLPLRFGLVIKTWESVTEQLITPYKTQLKELFAKLSGQREVSIKIFWDNQW
GvpF (Hbt. salinarum)   EDVEAHNNVLQEVLKHEEER---TVVPMSFGMAFKSA-RTLKGVLRGARRALRSTLNDIEGTVELGVKIL---GP
GvpL (Hbt. salinarum)   EQVKRWLVTHQQVVDAASDA-FGTPLPMRFDTVLEGGDASIERWLEDHYEGFRDELASFAGVWEYRINLLWDSAP

                                            II
GvpF (M. aeruginosa)    ELQAALESNPKLKQERDAMMGKNLNMEEIIHIGQLIEATVLRR------KQDIIQVFRDQLNHRAQEVIESDPMT
GvpF (Hbt. salinarum)   GDDTVP-------------------------------------------REEIQENVTDQLADLSINETENDLFT
GvpL (Hbt. salinarum)   FEETIADRDDRLRELRQRHNNRAAGKKFLLEKQSDQRLQELKRERRTELADQLKEAITPVVNDLTEQDTNTPLQD

GvpF (M. aeruginosa)    DDMIYNAAYLIPW------EQEPEFSQNVEAIDQQFGDRLRIRYNNLTAPYTFAQLI-----------
GvpF (Hbt. salinarum)   DRLIINKSYLVDF------EKRDAFDSAIDDVEAEY-DELTIQYTGPWPPYNFV-DIHIGAEQQQGGR
GvpL (Hbt. salinarum)   EHSSIEKEQIVRFAVLADEDDETALGDRLDTI-VEH-EGVEIRFTGPWPPYTFAPDI---------GK
```

B

```
                    α 1          β 1          β 2      α 2             α 3
pGvpM   MEPTKDETHAIVEFVDVLLRDGAVIQADVIVTVADIPLIGISLRAAIAGMTTMTEYGLFEEWDAAHRQQSEAFTTSPTADRRED
cGvpM   MNPTKRNSHAIVEFVDVLLRDGAVIQADAIVTVAGVPLLGISLRAAIAGMTTMTEYGIFDGWDADHRRRNAQP
```

Figure S2. Sequence alignment of GvpF and GvpL and of cGvpM and pGvpM. Identical amino acids are marked in yellow. **(A)** Sequence alignments of *Microcystis aeruginosa* and *Halobacterium salinarum* GvpF and of GvpL from *Hbt. salinarum*. Major structural differences are labelled with I and II (see also Fig. 7C). **(B)** Sequence alignment of pGvpM and cGvpM. The predicted secondary structure is indicated on top.

Table S1: Oligonucleotides used in this study

Name	Oligonucleotide Sequence (5‘ → 3‘)*
5’BspHI-NGFP	**TCATGA**GTAAAGGAGAAGAACTTTTCACTG
3’BlpI-link-NGFP	**GCTCAGC**GATTGGCCATGGCCGAGCCAGAGCCAGAGCCACCCGAGCCAGAGCCAGAGCCACCTTGTTTGTCTGCCGTG
5’NcoI-link-CGFP	**CCATGG**CTAATGCATGCAATGGGATCCTGGGTGGAAGCGGTGGTGGAAGCGGTGGTGGAAGCGGTGGTGGAAGCGGTAAGAATGGAATCAAAGCTAACTTCAAAATTAG
3’KpnI-CGFP	ATTATT**GGTACC**TTATTTGTATAGTTCATCCATGCCATGTGTAATCC
5’NcoI/BlpI-link-NIIb	**CCATGG**CCAATC**GCTGAGC**GGTGGCTCTGGCTCTGGCTCGGGTGGCTCTGGCTCTGGCTCGGGGATGAGTAAAGGAGAAGAACTTTTCACTGGA
3’KpnI-NGFPIIb	**GGTACC**TCATTGTTTGTCTGCCGTGATGTATACGTTGTG
5’NcoI-CGFPIIb	**CCATGG**ATGAAGAATGGAATCAAAGCTAACTTCAAAATTAGACACAACATTG
3’BamHI/KpnI-CIIb	**GGTACC**ATTGCATGCATTAG**GGATCC**ACCGCTTCCACCACCGCTTCCACCACCGCTTCCACCACCGCTTCCACCTTTGTATAGTTCATCCATGCCATGTGTAATCCC
5’NcoI-pM	GCGCTA**CCATGG**AGCCAACAAAAGACG
3’BlpI-pM	TGTTGTT**GCTGAGC**TCAGTCCTCTCGCCG
3’BlpI-pMΔStop	TGTTGTT**GCTCAGC**GAGTCCTCTCGCCG
5’BamHI-pM	AGGTCAA**GGATCC**ATGGAGCCAACAAAAG
3’KpnI-pM	AGTTCT**GGTACC**TCAGTCCTCTCGCCGATC
3’BamHI-pMΔStop	AGTTCT**GGATCC**CGTCCTCTCGCCG
5’BspHI-pL	TGTTGTT**TCATGA**CTGACCACCGGCC
3’BlpI-pL	TGTTGTT**GCTCAGC**TTATTTACCAATATCTGGCG
3’BlpI-pLΔStop	TGTTGTT**GCTCAGC**GATTTACCAATATCTGGCG
5’BamHI-pL	AGGTCAA**GGATCC**ATGACTGACCACCG
3’KpnI-pL	AGTTCT**GGTACC**TTATTTACCAATATCTGGCGCG
3’BamHI-pLΔStop	AGTTCT**GGATCC**CTTTACCAATATCTGGCGC
5’BspHI-pF	ACACGA**TCATGA**CTGAGAACCTATACACATACGGTATCATC
3’KpnI-pF	AGTTCT**GGTACC**TTATCGGCCTCCTTGTTGCTGTTC
3’BlpI-pFΔStop	TGTTGTT**GCTCAGC**GATCGGCCTCCTTGTTGCTGTTC
5’BamHI-pF	AGGTCAA**GGATCC**ATGACTG AGAACCTATACACATACG
3’KpnI-pF	AGTTCT**GGTACC**TTATCGGCCTCCTTGTTGCTGTTC
3’BamH-pFΔStop	AGGTCAA**GGATCC**CTCGGCCTCCTTGTTGCTGTTC
5’NcoI-pH	ACACGA**CCATGG**TTCCCGACGAAAACGACGA
3’KpnI-pH	ATTC**GGTACC**TCATGTGGATTCACCTCC
3'Bsu63I-pHΔStop	AGGTCA**CCTCAGG**GATGTGGATTCACCTCCATCGGTG
5'BamHI-pH	AATTAA**GGATCC**ATGGTTCCCGACGAAAACG
3'KpnI-pH	**GGTACC**TCATGTGGATTCACCTCCATCG
3'BamHI-pHΔStop	AATTAA**GGATCC**CTGTGGATTCACCTCCATC
5'BspHI-pJ	ACTGAC**TCATGA**GTGACCCCAAACCG

Name	Oligonucleotide Sequence (5‘ → 3‘)*
3’BlpI-pJ	GTAACCT**<u>GCTCAGC</u>**TCATTTGGTCTCCTCCG
3’BlpI-pJΔStop	AAGTAACCT**<u>GCTCAGC</u>**CCTTTGGTCTCCTCCGC
5'BamHI-pJ	AGGTCAA**<u>GGATCC</u>**ATGAGTGACCCCAAAC
3’KpnI-pJ	**<u>GGTACC</u>**TCATTTGGTCTCCTCCGCTG
3'BamHI-pJΔStop	TGGATT**<u>GGATCC</u>**CTTTGGTCTCCTCCGC
fwd-pMN25-pNGFPIIa	TGAGCTGAGCCGCG
rev-pMN25	AATCACGGCTCCGTCGC
fwd-pMN25-pNGFPIIb	TCGCTGAGCGGTGGC
fwd-pMN25-pCGFPIIb	TGAGGTACCGCGTGTGAAG
fwd-pMN25-pCGFPIIa	GGGATCCTGGGTGGAAG
M(25-59) X pNIIa-fwd	CATGATTCAAGCGGACGTGATCGTGACGGTCGCCGACATTCCCCTGATCGGGATCAGCCTCCGGGCAG CGATTGCTGGCATGACCACCATGACGGAGTACGGCCTGTTCTAAGGTAC
M(25-59) X pNIIa-rev	CTTAGAACAGGCCGTACTCCGTCATGGTGGTCATGCCAGCAATCGCTGCCCGGAGGCTGATCCCGATC AGGGGAATGTCGGCGACCGTCACGATCACGTCCGCTTGAAT
M(25-59) X pNIIb-fwd	CATGATTCAAGCGGACGTGATCGTGACGGTCGCCGACATTCCCCTGATCGGGATCAGCCTCCGGGCAG CGATTGCTGGCATGACCACCATGACGGAGTACGGCCTGTTCTCGC
M(25-59) X pNIIb-rev	TCAGCGAGAACAGGCCGTACTCCGTCATGGTGGTCATGCCAGCAATCGCTGCCCGGAGGCTGATCCCG ATCAGGGGAATGTCGGCGACCGTCACGATCACGTCCGCTTGAAT
M(25-59) X pCIIb-fwd	GATCCATTCAAGCGGACGTGATCGTGACGGTCGCCGACATTCCCCTGATCGGGATCAGCCTCCGGGCA GCGATTGCTGGCATGACCACCATGACGGAGTACGGCCTGTTCTAAGGTAC
M(25-59) X pCIIb-rev	CTTAGAACAGGCCGTACTCCGTCATGGTGGTCATGCCAGCAATCGCTGCCCGGAGGCTGATCCCGATC AGGGGAATGTCGGCGACCGTCACGATCACGTCCGCTTGAATG
M(25-59) X pCIIa-fwd	CATGATTCAAGCGGACGTGATCGTGACGGTCGCCGACATTCCCCTGATCGGGATCAGCCTCCGGGCAG CGATTGCTGGCATGACCACCATGACGGAGTACGGCCTGTTCGG
M(25-59) X pCIIa-rev	GATCCCGAACAGGCCGTACTCCGTCATGGTGGTCATGCCAGCAATCGCTGCCCGGAGGCTGATCCCGA TCAGGGGAATGTCGGCGACCGTCACGATCACGTCCGCTTGAAT
fwd-pMC25	GAGGAGTGGGATGCTGC
rev-MC25-NGFPIIa	CATGGCCGAGCCAGAG
rev-MC25-NIIb/CIIa	CATGGGCATCACTGCAGAG
rev-MC25-CGFPIIb	CATGGATCCACCGCTTCC
5’NcoI-pMΔ5N	GCGCTA**<u>CCATGG</u>**ACGAGACACACG
5'BspHI-MΔ10N	GCATCG**<u>TCATGA</u>**TCGTTGAGTTCGTCG
3’BamHI-MΔ10C-Stop	AGGTCA**<u>GGATCC</u>**CCGTGAACGCTTCGCTC
3’BamHI-MΔ20C-Stop	AGGTCA**<u>GGATCC</u>**CAGCATCCCACTCCTCG
3’BamHI-MΔ27C-Stop	AGGTCA**<u>GGATCC</u>**CGCCGTACTCCGTCATG
3’BamHI-MΔ30C-Stop	AGGTCA**<u>GGATCC</u>**CCGTCATGGTGGTCATGC

* Recognition sites for restriction enzymes are underlined and bold.

Table S2: Quantification of the fluorescence of the transformants investigated in this study. The averaged LAU/mm^2 values, the standard deviation and relative fluorescence (rf) are given. The standard deviation is calculated from two biological samples and three technical replicates each.

transformant		LAU/mm^2	σ (LAU/mm^2)	relative fluorescence (rf)
**controls**				
	WR340	14,303	2,615	0.00
	$_N$-/-$_C$	12,213	704	0.06
	$_N$-/$_C$-	11,981	919	0.05
	-$_N$/-$_C$	10,992	3,113	0.04
	-$_N$/$_C$-	10,898	839	0.02
M$_C$ /$_N$L	37°C	82,069	1,617	4.21
	30°C	185,342	29,656	12.13
**L-M interaction**				
$_N$M	L$_C$	26,483	9,310	0.96
	$_C$L	10,993	1,355	0.00
M$_N$	L$_C$	32,392	5,667	1.29
	$_C$L	10,289	1,222	0.00
$_C$M	L$_N$	11,026	1,266	0.00
	$_N$L	11,953	797	0.00
M$_C$	L$_N$	37,319	10,631	1.62
	$_N$L	185,342	29,656	12.13
$_N$M(25N)	L$_C$	425,472	65,574	26.27
	$_C$L	17,253	1,540	0.10
M(25N)$_N$	L$_C$	786,343	14,674	50.91
	$_C$L	21,767	1,830	0.32
$_C$M(25N)	L$_N$	13,519	1,382	0.00
	$_N$L	20,296	1,703	0.21
M(25N)$_C$	L$_N$	353,172	42,247	20.94
	$_N$L	590,416	115,683	38.18
$_N$M(25-59)	L$_C$	30,379	2,288	0.67
	$_C$L	19,640	2,220	0.12
M(25-59)$_N$	L$_C$	18,465	1,611	0.02
	$_C$L	16,313	4,884	0.00
$_C$M(25-59)	L$_N$	19,861	1,526	0.05
	$_N$L	24,615	1,968	0.37
M(25-59)$_C$	L$_N$	21,117	1,580	0.04
	$_N$L	35,829	1,427	0.92
$_N$M(25C)	L$_C$	265,443	35,073	16.06
	$_C$L	15,873	2,437	0.00
M(25C)$_N$	L$_C$	39,642	2,949	1.50
	$_C$L	12,250	1,247	0.00
$_C$M(25C)	L$_N$	11,671	1,986	0.00
	$_N$L	14,068	1,124	0.00
M(25C)$_C$	L$_N$	13,517	1,624	0.02
	$_N$L	14,198	1,852	0.00
**F-M interaction**				
$_N$M	F$_C$	14,776	5,568	0.13
	$_C$F	10,498	1,763	0.00
M$_N$	F$_C$	14,948	1,066	0.03
	$_C$F	11,339	1,497	0.00

transformant		LAU/mm^2	σ (LAU/mm^2)	relative fluorescence (rf)
$_C$M	F$_N$	10,352	1,278	0.00
	$_N$F	11,404	2,402	0.01
M$_C$	F$_N$	38,255	2,072	1.57
	$_N$F	38,253	1,829	1.58
$_C$M	F$_N$	10,352	1,278	0.00
	$_N$F	11,404	2,402	0.01
M$_C$	F$_N$	38,255	2,072	1.57
	$_N$F	38,253	1,829	1.58
$_N$M(25N)	F$_C$	42,119	8,088	1.82
	$_C$F	11,039	614	0.00
M(25N)$_N$	F$_C$	29,362	3,970	0.96
	$_C$F	11,015	1,613	0.00
$_C$M(25N)	F$_N$	10,082	1,223	0.00
	$_N$F	11,325	862	0.00
M(25N)$_C$	F$_N$	40,464	3,892	1.74
	$_N$F	37,007	2,709	1.50
$_N$M(25-59)	F$_C$	17,918	1,901	0.12
	$_C$F	13,271	962	0.00
M(25-59)$_N$	F$_C$	19,292	1,706	0.19
	$_C$F	14,808	2,122	0.01
$_C$M(25-59)	F$_N$	13,566	2,467	0.00
	$_N$F	14,529	1,693	0.02
M(25-59)$_C$	F$_N$	19,842	1,359	0.23
	$_N$F	18,461	1,898	0.15
$_N$M(25C)	F$_C$	192,400	6,175	11.94
	$_C$F	16,655	806	0.12
M(25C)$_N$	F$_C$	24,720	4,525	0.66
	$_C$F	10,783	2,103	0.00
$_C$M(25C)	F$_N$	10,301	1,402	0.00
	$_N$F	11,766	714	0.00
M(25C)$_C$	F$_N$	10,982	2,200	0.00
	$_N$F	10,275	2,295	0.00
**H-M interaction**				
$_N$M	H$_C$	15,652	1,624	0.00
	$_C$H	12,316	2,266	0.00
M$_N$	H$_C$	12,194	1,492	0.00
	$_C$H	10,535	1,085	0.00
$_C$M	H$_N$	11,000	1,509	0.00
	$_N$H	10,828	1,243	0.00
M$_C$	H$_N$	16,032	1,702	0.00
	$_N$H	12,622	1,466	0.00
$_N$M(25N)	H$_C$	27,547	1,474	1.47
	$_C$H	9,120	620	0.00
M(25N)$_N$	H$_C$	19,479	813	0.75
	$_C$H	10,159	527	0.00
$_C$M(25N)	H$_N$	9,713	1,125	0.01
	$_N$H	11,744	943	0.07
M(25N)$_C$	H$_N$	17,799	1,429	0.60
	$_N$H	15,739	653	0.41

transformant		LAU/mm²	σ (LAU/mm²)	relative fluorescence (rf)
$_N$M(25-59)	H$_C$	9,946	712	0.00
	$_C$H	9,144	336	0.00
M(25-59)$_N$	H$_C$	11,998	655	0.07
	$_C$H	10,977	887	0.02
$_C$M(25-59)	H$_N$	9,636	1,858	0.04
	$_N$H	10,992	484	0.01
M(25-59)$_C$	H$_N$	12,500	1,017	0.10
	$_N$H	11,561	595	0.03
$_N$M(25C)	H$_C$	156,171	2,265	11.79
	$_C$H	14,047	558	0.15
M(25C)$_N$	H$_C$	21,712	1,066	0.78
	$_C$H	10,925	417	0.00
$_C$M(25C)	H$_N$	11,406	1,160	0.05
	$_N$H	11,201	641	0.00
M(25C)$_C$	H$_N$	11,273	1,006	0.03
	$_N$H	11,542	915	0.00
**J-M interaction**				
$_N$M	J$_C$	8,755	2,314	0.34
	$_C$J	9,020	2,700	0.34
M$_N$	J$_C$	8,095	2,276	0.24
	$_C$J	8,972	3,021	0.32
$_C$M	J$_N$	9,939	2,021	0.37
	$_N$J	8,183	2,823	0.41
M$_C$	J$_N$	9,330	2,548	0.40
	$_N$J	9,864	2,152	0.51
$_N$M(25N)	J$_C$	15,789	1,493	0.57
	$_C$J	10,935	1,523	0.10
M(25N)$_N$	J$_C$	15,022	963	0.49
	$_C$J	11,296	1,514	0.15
$_C$M(25N)	J$_N$	12,195	1,030	0.21
	$_N$J	11,355	1,840	0.14
M(25N)$_C$	J$_N$	16,946	951	0.68
	$_N$J	16,685	1,969	0.66
$_N$M(25-59)	J$_C$	14,979	700	0.11
	$_C$J	14,562	2,147	0.14
M(25-59)$_N$	J$_C$	16,121	1,629	0.18
	$_C$J	16,362	2,723	0.24
$_C$M(25-59)	J$_N$	16,047	1,353	0.18
	$_N$J	15,864	2,278	0.20
M(25-59)$_C$	J$_N$	16,970	987	0.24
	$_N$J	17,729	3,088	0.32
$_N$M(25C)	J$_C$	47,920	3,298	1.91
	$_C$J	17,011	1,595	0.05
M(25C)$_N$	J$_C$	20,220	2,125	0.23
	$_C$J	15,259	8,867	0.00
$_C$M(25C)	J$_N$	16,423	1,561	0.03
	$_N$J	15,530	622	0.00
M(25C)$_C$	J$_N$	17,799	834	0.08
	$_N$J	16,396	458	0.01

transformant		LAU/mm²	σ (LAU/mm²)	relative fluorescence (rf)
**L/F + M deletion variants**				
$_N$L/M(mut)$_C$	Δ5N	109,188	16,180	5.54
	Δ10N	51,699	15,351	1.82
	Δ10C	161,133	8,823	9.18
	Δ20C	181,034	24,615	8.68
	Δ27C	177,317	36,296	8.80
	Δ30C	150,920	40,532	6.89
$_N$F/M(mut)$_C$	Δ5N	40,649	2,619	1.81
	Δ10N	21,915	894	0.52
	Δ10C	28,124	3,618	0.95
	Δ20C	28,97	2,701	1.00
	Δ27C	26,556	1,099	0.84
	Δ30C	21,581	924	0.49
